# *Komagataeibacter intermedius* V-05: An Acetic Acid Bacterium Isolated from Vinegar Industry, with High Capacity for Bacterial Cellulose Production in Soybean Molasses Medium

**DOI:** 10.17113/ftb.59.04.21.7148

**Published:** 2021-12

**Authors:** Rodrigo José Gomes, Paula Cristina de Sousa Faria-Tischer, Cesar Augusto Tischer, Leonel Vinicius Constantino, Morsyleide de Freitas Rosa, Roberta Torres Chideroli, Ulisses de Pádua Pereira, Wilma Aparecida Spinosa

**Affiliations:** 1 Department of Food Science and Technology, State University of Londrina, Celso Garcia Cid (PR 445) Road, 86057-970 Londrina, PR, Brazil; 2 Department of Biochemistry and Biotechnology, State University of Londrina, Celso Garcia Cid (PR 445) Road, 86057-970 Londrina, PR, Brazil; 3 Department of Chemistry, State University of Londrina, Celso Garcia Cid (PR 445) Road, 86057-970 Londrina, PR, Brazil; 4 Embrapa Agroindústria Tropical, 2270 Dra. Sara Mesquita Road, 60511-110 Fortaleza, CE, Brazil; 5 Department of Veterinary Preventive Medicine, State University of Londrina, Celso Garcia Cid (PR 445) Road, 86057-970 Londrina, PR, Brazil

**Keywords:** bacterial isolation, acetic fermentation, soybean co-product, microbial polysaccharide, physicochemical characterization

## Abstract

**Research background:**

Despite the great properties of bacterial cellulose, its manufacture is still limited due to difficulties in large-scale production. These problems are mainly related to low production yields and high overall costs of the conventional culture media normally used. To surpass these problems, it is necessary to identify new cheap and sustainable carbon sources. Thus, this work aims to isolate and select a high cellulose-producing *Komagataeibacter* strain from vinegar industry, and study its potential for bacterial cellulose synthesis in an industrial soybean co-product, known as soybean molasses, used as fermentation medium.

**Experimental approach:**

One isolated strain was able to produce high amount of cellulose in the standard Hestrin-Schramm medium, so we tested its ability to produce this biopolymer in a soybean molasses medium. The characteristics and properties of the produced bacterial cellulose membranes were analyzed by thermogravimetric analysis, X-ray diffraction, infrared spectroscopy, water-holding capacity and rehydration ratio. Genetic analysis of the selected strain served to determine its genus and species.

**Results and conclusions:**

An isolated strain that produced the highest amount of cellulose in Hestrin-Schramm medium (3.7 g/L) was genetically identified as *Komagataeibacter intermedius* V-05. This strain produced 10.0 g/L of cellulose in soybean molasses medium. Membranes from both substrates had similar chemical structure, crystallinity and thermal degradation. Soybean molasses proved to be a suitable alternative medium for biosynthesis of cellulose in comparison with the standard medium. In addition to providing higher production yield, the membranes showed great structural characteristics, similar to those obtained from standard medium.

**Novelty and scientific contribution:**

In this research, we have isolated and identified a *Komagataeibacter* strain which exhibits a high capacity for cellulose production in soybean molasses. The isolation and selection of strains with high capacity for microbial metabolite production is important for decreasing bioprocess costs. Furthermore, as there is a necessity today to find cheaper carbon sources to obtain microbial products at a lower cost, soybean molasses represents an interesting alternative medium to produce bacterial cellulose for its industrial application.

## INTRODUCTION

Acetic acid bacteria (AAB) belong to the family *Acetobacteraceae*, which includes several genera and species. They are strictly aerobic, Gram-negative or Gram-variable, catalase-positive, ellipsoidal to rod-shaped cells that can occur as individuals, or in pairs or chains. AAB are involved in the production of several products, such as vinegar, kombucha, gluconic acid, sorbose and bacterial cellulose (BC). This group of bacteria is described as nutritionally demanding microorganisms, and is difficult to isolate and cultivate in artificial media, particularly the strains isolated from environments with high concentrations of acetic acid (*1*).

Bacterial cellulose is an extracellular polysaccharide consisting of a chain of β-(1→4) linked d-glucose units, secreted from various species of bacteria. The most common and attractive strain is *Komagataeibacter xylinus*, a member of the family *Acetobacteraceae*, due to its ability to produce large amounts of cellulose and to consume a variety of sugars and other compounds as carbon sources. In recent years, the microbiological production of cellulose has attracted interest due to the unusual properties and characteristics of BC. Unlike cellulose synthesized by plants, which normally contains impurities, BC is free of lignin, hemicellulose, pectin and proteins, is highly organized and crystalline, with a huge water-absorbing and -holding capacities, and has interesting physical properties, such as higher tensile strength and elasticity. The excellent biocompatibility and biodegradability of BC also plays an important role in its use as prosthetic tissue (*1*, *2*).

High productivity in microbial processes typically depends on the isolation and selection of highly producing microorganisms, and their cultivation in an efficient culture medium. The nutrient source of a fermentation medium influences the growth and metabolism of cells. Therefore, the productivity of a fermentation process is strongly influenced by the culture medium composition, including the carbon and nitrogen sources, growth factors and inorganic salts (*3*). Carbon and nitrogen sources are the highest cost components of the medium for any fermentation process. As the problems associated with bioprocesses are typically productivity and production cost, there is a need to develop and use low-cost carbon sources, in order to produce microbial metabolites at a lower cost and higher production yield for industrial applications (*4*, *5*). The Hestrin-Schramm (HS) is a common and effective medium for BC production, but is limited in its ability to obtain high productivity in a large-scale production system due to its high cost (*6*). Thus, many studies have been dedicated to finding alternative substrates with lower cost. Agricultural residues such as fruit and vegetal peels (*7*, *8*), milk whey (*4*, *9*), and beet molasses (*9*) have been shown to be promising alternative media.

Soybean molasses is an industrial co-product, generated in the production of soy protein concentrate from defatted soybean meal. It is obtained by ethanol precipitation of soybean proteins, resulting in a brown syrup containing a high percentage of carbohydrates (approx. 70% soluble solids), as well as lipids, proteins, fiber and minerals. A large quantity of this by-product is used for animal feed or discarded (*10*). However, due to its composition, the soybean molasses can also be used as a fermentation medium to obtain microbial metabolites and products, such as bioethanol (*11*), vinegar (*10*) and exopolysaccharides (*12*).

In this work, we aim to isolate strains of AAB from vinegar industry, and evaluate the ability of BC synthesis using the soybean molasses as a carbon and nitrogen sources, followed by characterization of the BC produced in this substrate.

## MATERIALS AND METHODS

### Materials

Soybean molasses was supplied by Selecta soybean industry (Araguari, Brazil), in the concentrated form (approx. 70 °Bx). Strains of acetic acid bacteria (AAB) were isolated from fruit vinegar broth obtained from the ’Tecnologia em Saúde’ food industry (Assis, Brazil). Yeast extract, peptone and bacteriological agar were purchased from HiMedia (Mumbai, India). Other chemical reagents used for production of culture media were purchased from Synth (Diadema, Brazil).

### Isolation of the microorganisms

The culture media used for isolation were MYP (25 g/L mannitol, 5 g/L yeast extract and 3 g/L peptone) and Hestrin-Schramm (HS; 20 g/L glucose, 5 g/L yeast extract, 5 g/L peptone, 2.7 g/L Na_2_HPO_4_ and 1.15 g/L citric acid). Aliquots of fermenters were transferred to flasks containing the media described above, and incubated at (30±0.5) °C and 120 rpm for 96 h. When turbidity occurred, 0.1 mL of each diluted medium was transferred to Petri dishes containing a double layer of mannitol yeast extract peptone (MYP) agar (addition of a 0.5% agar layer and a 1.0% agar overlay) (*13*). Pure colonies with similar morphological characteristics were obtained using the streak plate method. The selected colonies were re-streaked several successive times until a pure culture was obtained. After purification, the well-isolated colonies were submitted for Gram staining to select Gram-negative and rod-shaped bacteria.

### Biochemical characterization

Oxidation of ethanol to acetic acid was investigated by colony inoculation in Petri dishes containing Carr medium (30 g/L yeast extract, 20 g/L ethanol, 0.022 g/L bromocresol green and 10 g/L agar). In this medium, the oxidation of ethanol generates acetic acid and changes the color from green to yellow. Overoxidising capacity was measured by the return of green color after extended incubation period (*1*). Oxidation of lactate was observed in dextrose sorbitol mannitol (DSM) agar (1 g/L glucose, 1 g/L sorbitol, 2 g/L mannitol, 3.3 g/L yeast extract, 10 g/L proteose peptone, 15 g/L calcium lactate, 1 g/L KH_2_PO_4_, 0.02 g/L MnSO_4_·H_2_O, 0.03 g/L bromocresol purple, 0.29 μg/L brilliant green and 15 g/L agar). This medium changes the color from yellow to purple, as a result of lactate utilization, causing a pH increase (*1*). Production of catalase was observed after the addition of 3% (*V*/*V*) hydrogen peroxide (Anidrol, Diadema, Brazil) on bacterial colonies grown in MYP agar. Bubble formation indicated the presence of catalase. Production of indole from tryptophan was observed after growth in tryptone-containing medium (10 g/L tryptone) and addition of 0.1 mL Kovac’s reagent (Probac, São Paulo, Brazil). Red color formed on the surface of the broth indicated a positive result, while yellow color indicated a negative result. Ketogenic activity was detected by covering an inoculated agar plate (30 g/L yeast extract, 30 mL/L glycerol and 20 g/L agar) with Fehling's solution (Anidrol). Around positive colonies, an orange-colored halo of CuO was formed. Production of cellulose is described in a separate section below. The strain exhibiting the highest cellulose production in this step was selected for further experiments, including genetic characterization, biosynthesis of bacterial cellulose (BC) in soybean molasses medium, and characterization of the produced BC.

### Extraction and amplification of DNA

For genetic characterization, DNA from an isolated strain was extracted using the PureLink^TM^ Genomic DNA Mini Kit (Invitrogen, Thermo Fisher Scientific, Eugene, OR, USA). Extracted DNA was amplified using the Platinum^TM^ PCR SuperMix kit (Invitrogen, Thermo Fisher Scientific), according to the following conditions: 45 µL of each reaction containing PCR SuperMix (Thermo Fisher Scientific, Waltham, USA), 1 µL of each primer (10 nM) and 3 µL of DNA template (approx. 50 ng). All products were analyzed by electrophoresis in a 2% agarose gel with Safer dye (Kasvi, São José dos Pinhais, Brazil) in 0.5X TBE buffer (89 mM Tris, 89 mM boric acid, 2 mM EDTA; Thermo Fisher Scientific), pH=8.4, and visualized under ultraviolet light. Molecular size was estimated by comparison with a 100 bp ladder.

### Molecular typing of isolated strain

Amplification and sequencing of the 16S rRNA gene were performed for the selected strain in a randomized manner (*14*). The products of 16S rRNA gene amplification were purified with a PureLink^TM^ Genomic DNA extraction kit (Invitrogen, Thermo Fisher Scientific), quantified using a Qubit^TM^ 2.0 fluorometer (Invitrogen, Thermo Fisher Scientific), and sequenced on an ABI3500 genetic analyzer (Applied Biosystems, Foster City, CA, USA) using primers fD1 (5’- CCGAATTCGTCGACAACAGAGTTTGATCCTGGCTCAG-3’) and rD1 (5’-CCCGGGATCCAAGCTTAAGGAGGTGATCCAGCC-3’). The contigs were obtained by CAP3, and sequence quality was analyzed visually in BioEdit software v. 7.2.5 (*15*). The identity was compared with all sequences deposited in the non-redundant database of GenBank using the BLAST program (*16*). The alignment was created in the BioEdit program using the ClustalW package (*17*). The phylogenetic tree was built using the maximum likelihood method (*18*) and the MEGA v. 7.0.18 program (*19*).

### Characterization of soybean molasses

Moisture content was determined by drying in an oven (model EL 1.5; Odontobrás, Ribeirão Preto, Brazil) at 105 °C until constant dry mass. Crude protein was quantified on the basis of total nitrogen content (conversion factor equal to 6.25) by Kjeldahl method (Kjeltec 8400^TM^; Foss, Hillerød, Denmark). Total ash was determined by heating at 550 °C in a muffle furnace (model Q318M24; Quimis, Diadema, Brazil). Lipid content was determined by the solvent extraction method using chloroform/methanol/water 2:2:1.8 (Anidrol) (*20*). The content and composition of carbohydrates were determined by high performance liquid chromatography (HPLC), using a liquid chromatograph (model LC-10A; Shimadzu, Tokyo, Japan) coupled with a refractive index detector (RID-10A; Shimadzu). The samples were diluted and filtered through a 0.22 μm poly(vinylidene fluoride (PVDF) membrane (Millex®, Merck Millipore, Dublin, Ireland). The elution was performed on Aminex HPX-87P HPLC column (300 mm×7.8 mm, 9.0 μm i.d.; Bio-Rad, Hercules, CA, USA), in an isocratic system using ultrapure water as mobile phase, at a flow rate of 1.0 mL/min. Sugars were quantified by HPLC using known concentrations of standards of glucose, fructose, galactose, sucrose, raffinose, stachyose and xylose (Sigma-Aldrich, Merck, St. Louis, MO, USA). The content of fiber (in %) was estimated by subtracting the content of carbohydrates, proteins, lipids and ash from the total dry matter.

The concentrations of elements Mg, Co, Cr, Cu, B, Mn, Fe, Zn and Mo were determined by inductively coupled plasma mass spectrometry (ICP-MS, model 820-MS; Varian, Mulgrave, Australia). Plasma was generated using Ar. The main flow rate was 17 L/min and the nebulizer flow rate was 0.21 L/min. Concentration of Ca was determined using an atomic absorption spectrophotometer (model AA-140; Varian), equipped with a deuterium and a single element (Ca) cathode lamp, operating at a wavelength of 422 nm at 4 mA. Concentration of K was determined using a flame photometer (model B462; Micronal, São Paulo, Brazil). Concentration of P was determined using a visible spectrophotometer (AJX-1600; Micronal) at a wavelength of 660 nm *via* the phosphomolybdate complex formation method.

### Production of cellulose in Hestrin-Schramm and soybean molasses media

Using the isolated and selected cellulose-producing strain, colonies were transferred to flasks containing sterile Hestrin-Schramm (HS) medium and incubated in a bacteriological incubator (BOD TE-391; Tecnal, Piracicaba, Brazil) for approx. 10 days at (30±0.5) °C. From this inoculum, 10 mL were transferred into a 500-mL Erlenmeyer flask, containing 90 mL sterile soybean molasses diluted at 20 °Bx (molasses 70 °Bx/water ratio=1:2.5) and 2% ethanol. The flasks were incubated in a bacteriological incubator (BOD TE-391; Tecnal) under static conditions for 14 days at (30±0.5) °C. Cellulose was produced in standard HS medium using a similar method, by transferring 10% of inoculum into a 500-mL Erlenmeyer flask containing sterile HS broth, and incubating for 14 days at (30±0.5) °C under static conditions in a bacteriological incubator (BOD TE-391; Tecnal). The cellulose produced on the surface of each medium was collected and heated in 1 M NaOH solution (Synth) at 80 °C for 30 min in water bath (model Q215M2; Quimis), then washed with distilled water until reaching neutral pH. The bacterial cellulose membranes obtained after the treatment were dried at 105 °C in a drying oven (model EL 1.5; Odontobrás) until achieving constant mass, to determine their yields expressed as dry mass.

### Determination of sugar content in soybean molasses during fermentation

Sugar content at the beginning and end of soybean molasses fermentation was measured by HPLC (model LC-10A; Shimadzu) under the same conditions as described in the section *Characterization of soybean molasses*. Then, the parameters of production yield and substrate conversion rate were determined as described below.

### Bacterial cellulose production rate and production yield

The efficiency of *K. intermedius* V-05 bacterial cellulose (BC) production was evaluated after 336 h of cultivation. Its production rate *r* (in g/(L·h)), production yield *Y* (in %) and substrate conversion ratio *w* (in %) were calculated, respectively, as follows (*21*):

*r*(BC)=*m*_BC_/(*V*·*t*) /1/

*Y*(BC)=((*m*_BC_/*V*)/(*S*_i_-*S*_f_))·100 /2/

*w*(substrate conversion)=((*S*_i_-*S*_f_)/*S*_i_)·100 /3/

where *S*_i_ is the initial substrate concentration (g/L), *S*_f_ is the residual substrate concentration (g/L), *m*_BC_ is the mass of produced BC (g), *V* is the reaction volume (L) and *t* is time of fermentation (h).

### Thermogravimetric analysis

Thermal stability of the bacterial cellulose membranes was determined by thermogravimetric analysis (TGA) using a thermal analyzer (model TGA-50; Shimadzu). The scans were ramped from 0 to 600 °C at a heating rate of 10 °C/min, under nitrogen atmosphere (50 mL/min). Derivative form of TGA (differential thermogravimetry; DTG) was obtained using the differential of TGA values. The TGA and DTG curves represented the mass variation as a function of temperature.

### X-ray diffraction

The X-ray diffraction (XRD) patterns of the BC membranes were obtained using an X-ray diffractometer (Panalytical X'Pert Pro MPD; Malvern, Almelo, Netherlands) and Cu Kα radiation (*λ*=1.5418 Å) at 40 kV and 30 mA. All assays were performed with scan speed of 1 °/min, analyzing the range of 5–40° (2*θ*). The degree of crystallinity was determined as described previously (*22*).

### Fourier-transform infrared spectroscopy

Infrared spectra were recorded using a spectrometer (model MB100; ABB Bomem Inc., Quebec, Canada) over the 4000–400 cm^-1^ range to determine the main bands that characterize the bacterial cellulose polymer. A total of 8 scans were taken for each sample produced in soybean molasses and HS medium. The spectra were collected in transmission mode, with a resolution of 1 cm^-1^.

### Water-holding capacity and rehydration ratio

For determination of water-holding capacity (WHC), wet BC samples were shaken quickly and weighed after removal from the storage recipient (*m*_wet_). The samples were then dried at 60 °C for 48 h in order to completely remove water, and weighed again (*m*_dry_). WHC of BC samples was calculated as follows (*23*):

WHC=(*m*_wet_-*m*_dry_)/*m*_dry_ /4/

The dried BC membranes (*m*_dry_) were immersed in distilled water until the mass of the rehydrated sample (*m*_rwet_) no longer increased (approx. 24 h). The rehydration ratio (RR) was calculated as follows (*24*):

RR=((*m*_rwet_-*m*_dry_)/(*m*_wet_-*m*_dry_))·100 /5/

### Statistical analysis

Results of maximum BC production, WHC and rehydration ratio of the BC samples were compared between both soybean molasses and HS media using the statistical software R (*25*). Data were expressed as mean values of triplicate experiments±standard deviation (S.D.).

## RESULTS AND DISCUSSION

### Biochemical and genetic characterization of bacteria isolated from vinegar industry

Table 1 shows the biochemical characteristics of the isolated microorganisms. After the isolation step and biochemical tests, seven strains belonging to the family *Acetobacteraceae* (*1*) were obtained from industrial vinegar fermentation broth using the isolation technique of double-layer agar. The cultivation on this agar plate, by adding 0.5% agar and coating with a 1% agar layer, is considered the most efficient isolation technique, as it provides a wet environment for the formation of acidifying bacterial colonies (*13*). After biochemical tests, the strains were revealed to be Gram-negative bacteria that were able to oxidize ethanol to acetic acid and cause further oxidation to CO_2_ and H_2_O, as well as oxidation of lactate and production of gluconic acid from glucose. All strains were able to produce catalase, while only five and three of them were able to produce cellulose and dihydroxyacetone from glycerol, respectively.

**Table 1:** Biochemical characterization of the strains isolated from vinegar samples

Characteristic	Strain						
V01	V02	V03	V04	V05	V06	V07
Gram-negative rods	+	+	+	+	+	+	+
Oxidation of ethanol to acetic acid	+	+	+	+	+	+	+
Oxidation of acetic acid to CO_2_ and H_2_O	+	+	+	+	+	+	+
Oxidation of lactate to CO_2_ and H_2_O	+	+	+	+	+	+	+
Catalase reaction	+	+	+	+	+	+	+
Gluconic acid production from glucose	+	+	+	+	+	+	+
Ketogenesis (dihydroxyacetone) from glycerol	-	-	+	-	-	+	+
Indole production from tryptophan	-	-	-	-	-	-	-
*γ*(cellulose)/(g/L)	2.2	1.9	2.4	1.9	3.7	0.0	0.0

**Fig. 1:**
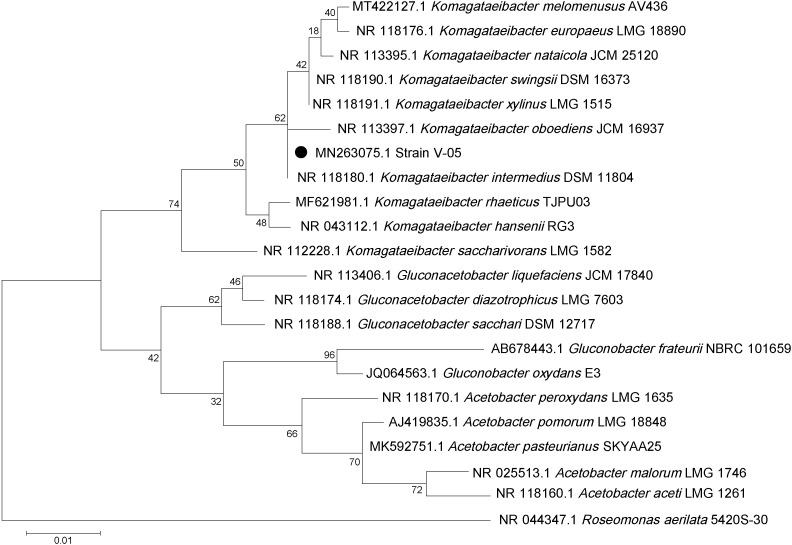
Phylogenetic tree of the strain V-05, representing its relative position in the genus *Komagataeibacter* based on 16S sequences. The maximum likelihood method was used for phylogenetic inference, and reliability of the nodes was estimated using 1000 bootstrap replicates. Evolutionary distances were computed using the Tamura-Nei method (*19*). The sequence obtained through sequencing was deposited in GenBank under the accession number MN263075.1

One strain preliminarily identified as V-05 showed the highest production of cellulose and was selected for genetic identification, and then for testing of BC production in soybean molasses medium. For this strain, the evolutionary history in the phylogenetic tree was inferred by using the maximum likelihood method (*18*), based on the Tamura-Nei model (*19*). According to Fig. 1, after genetic analysis of the amplified and sequenced 16S rRNA gene, the phylogenetic position of the selected strain V-05 was most closely related to the strain *Komagataeibacter intermedius* (GenBank accession no. MN263075.1). Through genetic and phylogenetic analysis, the strain V-05 was classified into the genus *Komagataeibacter* belonging to the species *intermedius*, an AAB first isolated and identified from vinegar samples and kombucha tea (*26*).

### Physicochemical characteristics of soybean molasses

Table 2 shows the results of soybean molasses physicochemical composition analysis, expressed on dry mass basis. Carbohydrates are the major components (approx. 70% *m*/*m*) of soybean molasses, mainly sucrose and smaller amounts of oligosaccharides (raffinose and stachyose), as well as monosaccharides (glucose, fructose, xylose and galactose). The most abundant sugars in soybean molasses, nearly 90% (*m*/*m*), are sucrose, raffinose and stachyose. However, among these sugars, only sucrose is naturally fermentable. As in soybeans, K, Ca, P and Mg are the main minerals present in soybean molasses (*27*). The high mass fraction of carbohydrates and other constituents, such as protein, lipids and minerals, makes soybean molasses an attractive substrate for use in fermentative processes. The source of carbon and nitrogen in the cultivation media usually plays an important role in fermentation productivity, as these nutrients are directly linked with the microbial biomass composition and the metabolic product formation (*28*). In addition, the microorganisms require various metals in ionic form, which are required in varying amounts for their growth. These ions play a biological role – for example, they act as enzyme activators (cofactors), regulate osmotic pressure, and participate in cellular respiration. The significant mass fraction of minerals in soybean molasses also contribute to the development of microorganisms involved in the fermentation. Mohite *et al*. (*29*) reported that the concentration of metal ions in the culture medium is an important factor for the production of BC, and also suggested that Mg and Ca have a positive effect on cellulose production.

**Table 2:** Physicochemical composition of soybean molasses on dry mass basis

Component	*w*(component)/(g/100 g)
Total carbohydrate Sucrose Raffinose Stachyose Glucose Fructose Galactose Xylose Protein Lipid Ash Fiber Potassium Calcium Phosphorus Magnesium Iron Boron Manganese Chromium Cobalt Copper Zinc Molybdenum	70.4±4.4 30.5±1.7 14.2±1.3 19.0±1.1 1.5±0.0 1.8±0.1 0.5±0.0 2.9±0.1 10.7±0.1 7.7±0.1 6.8±0.1 4.4±0.0 *w*(mineral)/(g/kg) 36.5±4.2 0.70±0.04 0.30±0.02 0.27±0.01 *w*(mineral)/(mg/kg) 0.31±0.06 0.03±0.00 0.02±0.00 0.01±0.00 n.d. n.d. n.d. n.d.

n.d.=not detected

### Production of bacterial cellulose

Strain V-05 showed high production rate and production yield when soybean molasses was used as fermentation substrate for BC production. The yield obtained in soybean molasses containing ethanol was approx. 3 times higher (10.0 g/L) than in standard HS medium (3.7 g/L), showing that soybean molasses is a potent substrate for BC biosynthesis. The maximum production of BC by *K. intermedius* V-05 in soybean molasses medium reached in this study was similar to that obtained by Vazquez *et al*. (*30*) using glycerol remaining from biodiesel production and corn steep liquor. Previously published results of BC obtained from complex medium and HS are compared to those from this work in Table 3 (*2*, *7*-*9*, *23*, *30*-*40*). Mean BC production rate and BC production yield were 0.0298 g/(L·h) and 34.25%, respectively. In preliminary tests, BC production by *K. intermedius* V-05 in soybean molasses without ethanol addition was much inferior (<1.0 g/L) to the production in the medium containing ethanol. It is known that the addition of ethanol at low volume fractions increases the production of cellulose. Li *et al*. (*42*) found that the production increased after the addition of ethanol to the used culture medium. Ethanol functions as an additional energy source for adenosine triphosphate generation at the early stage of fermentation by acting as a precursor for the growth of bacteria, and by allowing glucose to be used only for BC synthesis. Ethanol also functions as an electron donor (*42*). In this work, ethanol addition improved the production of BC in soybean molasses medium.

**Table 3:** Comparison of bacterial cellulose (BC) production from diverse complex media, by-products and agroindustrial wastes reported in literature

Complex medium	Strain	*t*/day	*γ*(BC)/(g/L)	*γ*(BC in HS)/(g/L)	Source
Carob and haricot bean	*Gluconacetobacter xylinus* ATCC 700178	10	3.2	-	(*31*)
Cashew apple juice and soybean molasses	*Gluconacetobacter xylinus* ATCC 53582	7	4.5	4.0	(*2*)
Cheese whey	*Gluconacetobacter xylinus* PTCC 1734	14	3.5	3.3	(*9*)
Citrus peel and pomace	*Komagataeibacter xylinus* CICC No. 10529	8	5.7	3.9	(*7*)
Cotton cloth hydrolysate	*Gluconacetobacter xylinus* ATCC 23770	14	10.8	5.9	(*32*)
Date syrup	*Acetobacter xylinum* 0416	10	5.8	~3.0	(*23*)
Grape bagasse and corn steep liquor	*Gluconacetobacter xylinus* NRRL B-42	14	8.0	~2.0	(*30*)
Orange juice	*Acetobacter xylinum* NBRC 13693	14	5.9	-	(*33*)
Overripe banana	*Komagataeibacter medellinensis NBRC 3288*	12	4.0	-	(*34*)
Pecan nutshell	*Gluconacetobacter entanii*	28	2.8	-	(*35*)
Pineapple peel juice	*Gluconacetobacter swingsii sp.*	13	2.8	2.1	(*36*)
Potato peel wastes	*Gluconacetobacter xylinum* ATCC 10245	6	4.7	1.2	(*8*)
Soybean molasses and ethanol	*Komagataeibacter intermedius* V-05	14	10.0	3.7	This work
Sugar beet molasses	*Gluconacetobacter xylinus* PTCC 1734	14	4.6	3.3	(*9*)
Waste beer yeast	*Gluconacetobacter hansenii* CGMCC 3917	14	7.0	-	(*37*)
Waste durian shell hydrolysate	*Gluconacetobacter xylinus*	10	2.7	2.5	(*38*)
Waste water from rice wine distillery	*Gluconacetobacter xylinus* BCRC 12334	7	6.3	~3.0	(*39*)
Waste glycerol and corn steep liquor	*Gluconacetobacter xylinus* NRRL B-42	14	10.0	~2.0	(*30*)
Waste water of candied jujube	*Gluconacetobacter xylinum* CGMCC No. 2955	6	2.2	-	(*40*)

HS=Hestrin-Schramm medium

**Fig. 2:**
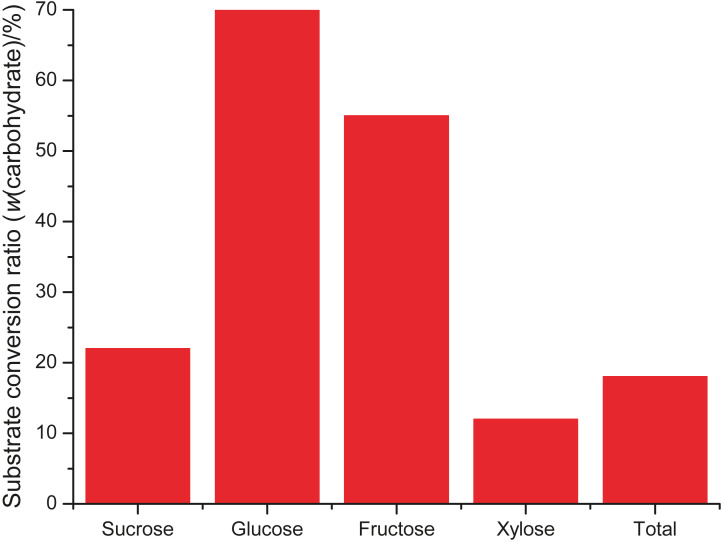
Consumption of sugars in soybean molasses after a 14 day-fermentation period

As shown in Fig. 2, the reducing sugars fructose and glucose were the main sugars consumed during fermentation, with 55 and 70% of the initial mass consumed per volume of medium, respectively. Raffinose and stachyose oligosaccharides are not used as a carbon source by AAB, as they do not produce α-galactosidase. An increase in the mass fraction of fermentable sugars and production yield may be achieved by enzymatic hydrolysis of the α-(1→6)-galactosidic bonds present in soybean molasses oligosaccharides, using α-galactosidase prior to fermentation (*11*). Low consumption of xylose is also observed in the fermentation for BC biosynthesis, attributed to the complexity in metabolizing this carbohydrate (*41*).

### Characterization of bacterial cellulose

**Fig. 3:**
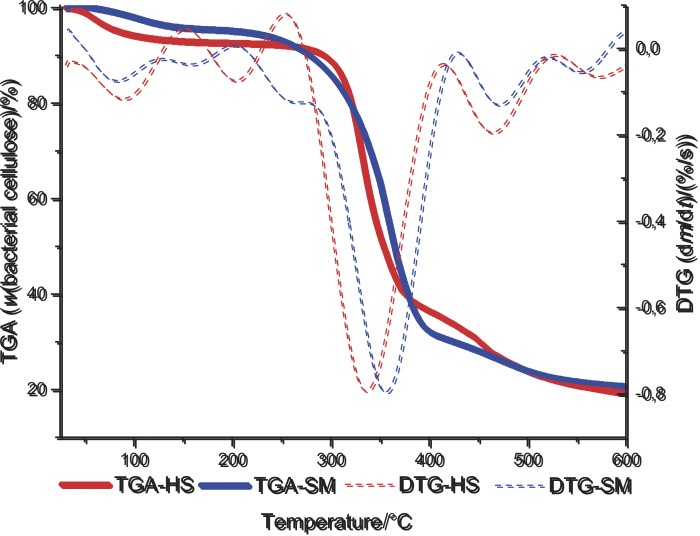
Thermogravimetric (TGA) and derivative thermogravimetric (DTG) curves of bacterial cellulose membranes produced by *K. intermedius* V-05 in soybean molasses (SM) and standard Hestrin-Schramm (HS) media

Thermal decomposition curves and the respective DTG curves of the bacterial cellulose membranes are shown in Fig. 3. From the obtained curves, showing mass percentage as a function of temperature, the parameters of initial thermal decomposition temperature (*t*_onset_), temperature where 10% of mass was lost (*t*_10%_), and mass loss at 600 °C were calculated. The membranes produced in HS medium exhibited higher parameters of *t*_onset_, *t*_10%_ and mass loss at 600 °C (*t*_onset_=307 °C and *t*_10%_=300 °C) than in soybean molasses medium (*t*_onset_=299 °C and *t*_10%_=285 °C). This fact may be correlated with the higher uniplanar properties and higher crystallinity index of these membranes, which require higher energy for their degradation. The membranes produced in HS medium also had higher mass loss at 600 °C (81%) than those from soybean molasses medium (79%).

Degradation curves of the BC membranes produced in soybean molasses medium presented similar and typical profiles, compared to when HS medium was used (Fig. 3). The downward peaks in DTG curves were consistent with the maximum mass loss of thermogravimetric curves. The maximum rate of mass loss was around 350 °C, similar to the reports in an earlier study (*34*). The thermal decomposition curves of the membranes indicated three distinct mass loss steps characteristic of BC process showing that it was stable up to 250 °C. The first mass loss was observed from room temperature (approx. 30 °C) to approx. 150 °C, and is attributed to mass loss due to evaporation of residual water resulting from drying. The second mass loss was observed in the temperature range from 250 to 400 °C and is attributed to degradation of cellulose (dehydration and decomposition of the glycosidic units). The third and final mass-loss extends up to 500 °C, corresponding to the thermo-oxidative degradation of cellulose (*43*). Earlier studies have reported a similar thermal stability and degradation temperature of bacterial cellulose as observed in this work (*2*).

**Fig. 4:**
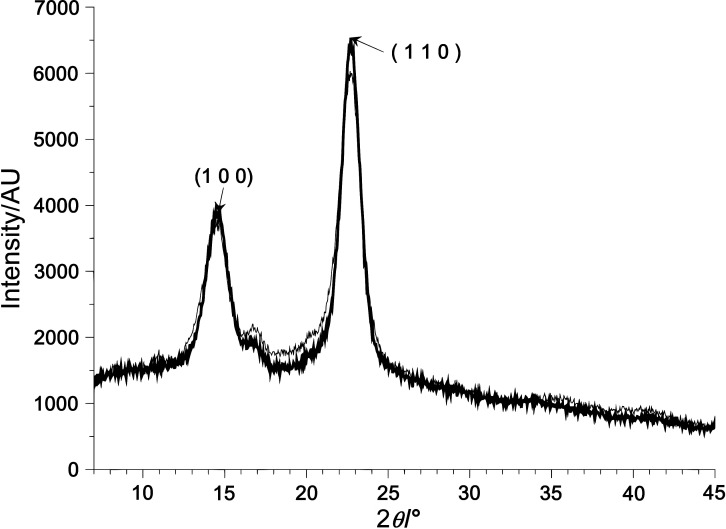
X-ray diffraction pattern of the cellulose membranes produced by *K. intermedius* V-05

As shown in Fig. 4, the XRD data demonstrated that the culture medium did not influence the crystal organization of the membranes. The membranes produced in both standard HS and soybean molasses with ethanol media showed similar crystalline profiles. The samples had two major peaks at 15 and 22.5°, assigned to the characteristic interplanar distances of cellulose Iα and Iβ phases (100Iα, 110Iβ and 010Iβ planes at 15° and 110Iα and 200Iβ at 22.5°) (*44*). The observed peaks in this work demonstrate that both BC samples possess typical crystalline forms of cellulose I. The samples produced in soybean molasses medium showed slightly higher (71.2%) crystallinity index than those from HS medium (69.0%). The values of crystallinity index observed in this work for the membranes produced in both media were similar to those reported by other authors in an earlier study (*45*).

**Fig. 5:**
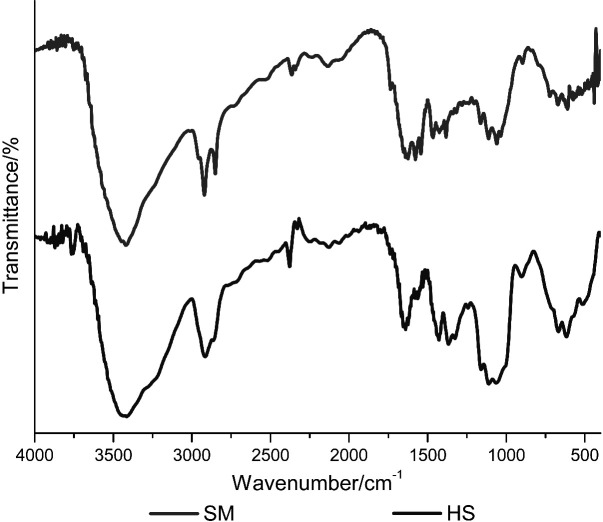
Fourier-transform infrared spectra of the cellulose membranes produced by *K. intermedius* V-05 in soybean molasses (SM) and standard Hestrin-Schramm (HS) media

Determination of functional groups of the BC samples was made by analyzing the spectra obtained at wavelengths from 400 to 4000 cm^-1^ by FTIR analysis. As shown in Fig. 5, the functional groups of the samples obtained from both fermentation media were almost the same. The FTIR spectra of both membranes displayed the main attributes that characterize the cellulose polymer, such as strong transmission of OH stretching vibrations at 3500 cm^-1^, alkane CH stretching and CH_2_ asymmetric stretching vibration at 2900 cm^-1^, CH_2_ symmetric stretching vibration at 2700 cm^-1^, OH deformation vibration at 1600 cm^-1^, CH_2_ deformation vibration at 1400 cm^-1^, CH_3_ deformation vibration at 1370 cm^-1^, OH deformation vibration at 1340 cm^-1^, and CO deformation vibration in the range of 1320–1030 cm^-1^ (*46*). Bands observed at 1640 cm^-1^ (*δ*s HOH) and 3500  cm^-1^ (*ν* OH) were attributed to water absorption by the composites, and the bands observed at 750 and 710 cm^-1^ correspond to phases Iα and Iβ, respectively, of the BC samples (*47*). The results of FTIR analysis are in agreement with studies carried out previously (*47*), indicating that the produced substance is chemically pure bacterial cellulose.

In relation to hydrophilic properties, BC membranes produced in soybean molasses medium exhibited lower WHC (approx. 57 times per dry mass) than those produced in HS medium (162 times per dry mass). Conversely, the soybean molasses with ethanol provided membranes with higher rehydration ratio than HS medium (4.4 and 1.2%, respectively). The WHC represents the water mass retained per unit of cellulose dry mass. This parameter is directly involved in biomedical applications of cellulose as a dressing material. Appropriate moisture content accelerates the wound healing process and protects against contamination. In addition, it facilitates the penetration of active substances into the wound, allowing more facile regeneration without damaging the newly formed skin (*48*). Several factors interfere with the hydrophilic properties of the BC membranes, such as the increase in the number of polar groups (*49*), and the drying method used, which can induce structural modifications, including roughness, shrinkage or collapse of some capillary structures (*50*). This affects the porosity and surface area, which can increase or reduce empty spaces among the BC fibrils, and thus, more or less water could penetrate and adsorb onto the material (*48*). WHC is a parameter related to wet sample of BC while rehydration ratio represents the degree to which removed water was recovered by the samples. Drying improves the storage and sell-life of BC, but poor rehydration capacity reduces the utility of the dried BC (*24*). In this work, it was observed that reduction of crystallinity caused an increase in WHC. This fact can be attributed to the larger number of amorphous regions in the structure, promoting water permeation into the cellulose network (*24*). Conversely, BC membranes produced in soybean molasses with ethanol exhibited higher rehydration ratio, which shows a higher recovery of the water removed during drying. Considering that this sample was thicker than those produced in HS medium, the increase of OH hydrophilic groups in the structure may have increased the water adsorption capacity, both on the surface and inside the cellulose matrix.

## CONCLUSIONS

In this work, a new cellulose-producing strain named *Komagataeibacter intermedius* V-05 was isolated from vinegar industry. Besides that, an alternative culture medium composed of soybean molasses supplemented with ethanol showed to be an attractive culture medium for the biosynthesis of bacterial cellulose (BC) by this strain. This complex medium provided higher production yield than the standard Hestrin-Schramm (HS) medium, the most commonly used medium for BC production. The bacterial cellulose membranes produced in soybean molasses with ethanol also presented the same crystallinity usually observed for BC, lower water-holding capacity and higher rehydration ratio than those produced in HS medium, demonstrating that the type of substrate used has an effect on the characteristics of the obtained cellulose.
